# The evaluation of the psychometric properties of a specific quality of life questionnaire for physiological pregnancy

**DOI:** 10.1186/1477-7525-11-214

**Published:** 2013-12-23

**Authors:** Eva Vachkova, Stanislav Jezek, Jiri Mares, Marketa Moravcova

**Affiliations:** 1 Division of Nursing, Department of Social Medicine, Charles University in Prague, Faculty of Medicine in Hradec Kralove, Prague, Czech; 2 Faculty of Social Studies, Institute for Research of Children, Youth and Family, Masaryk Univerzity, Brno, Czech; 3 Department of Social Medicine, Charles University in Prague Faculty of Medicine in Hradec Kralove, Prague, Czech; 4 Department of Midwifery and Health and Social Work, Faculty of Health Studies, University of Pardubice, Pardubice, Czech

**Keywords:** Pregnancy, Quality of life, Generic questionnaire, WHOQOL-BREF, Specific questionnaire, Pregnancy scale

## Abstract

**Background:**

Pregnancy is a specific condition that is neither a disease nor a normal state of health. The attention has been devoted to the relation between the normal, physiological process of pregnancy and the quality of life of women in this period is paid much less attention. Our study focuses on the evaluation of the quality of life by means of a specific questionnaire for physiological pregnancy. The main objective was to evaluate psychometric characteristics of a newly developed, specific QoL.

**Methods:**

Two measures were used: a Czech version of the generic WHOQOL-BREF, validated in 2006, and a new specific-QoL measure. Both measures were administered in each trimester to a sample of 225 pregnant women in the first trimester of a routine pregnancy.

**Results:**

The reliability of the WHOQOL-BREF scales at different trimesters was evaluated, including the correlation between trimesters. Based on exploratory factor analyses of the specific-QoL measure with the working title QOL-GRAV, one 9-item scale was constructed expressing the degree of specific experiences during pregnancy. All scales were found to have satisfactory internal consistency (Cronbach alphas > .7) apart from the social relations subscale of the WHOQOL-BREF.

**Conclusions:**

The general quality and the specific quality of a pregnant woman’s life varies. The specific QOL-GRAV scale is more sensitive to the specific experiences during pregnancy that significantly affect a pregnant woman’s quality of life. A simple specific questionnaire, applicable within prenatal care as well, was designed and validated.

## Background

Pregnancy is a specific condition that is not a disease or a normal state of woman’s health. Forger et al. point out that during pregnancy there are specific organ and hormonal changes that affect bodily functions and often the overall well-being of pregnant women. It results in changes to a pregnant woman’s quality of life [1].

At present, a number of research papers and articles try to define the concept of quality of life by dealing with its various aspects or areas e.g. [2-4].

Barofsky attributes the difficulty in defining quality of life to the absence of a method that could be used to define the quality of life and that would not have any limitations. We must bear in mind that the definitions of a comprehensive concept of quality of life are continually changing [5].

The study of the quality of life reflects the methodological diversity of scientific inquiry, the quantitative approach being the one most often used (see Table 1). It is represented by either the use of standardized generic tools or by evaluating the reliability and validity of newly modified tools, usually with respect to measures like the SF-36 e.g. [6]. In the last decade the research of the quality of life grew steadily under the umbrella title *Health-Related Quality of Life* (HRQL). Vachkova and Mares remark that the interest of researchers is focused more on difficulties, problems, and pathological aspects of pregnancy that adversely affect quality of life, e.g. the varying severity of nausea and vomiting (see Table 1). The relation between the normal, physiological process of pregnancy and a woman’s quality of life in this period is given much less attention [7].

**Table 1:** Questionnaire methods used abroad for assessing the quality of life of pregnant women (Taken from: Vachkova, Mares, 2012, p. 289)

**Research problem**	**Tool/Instrument**	**Authors**
**Nausea and vomiting**	Nausea Vomiting Pregnancy QOL (NVP QOL)	[8] Canada
	Pregnancy Unique Questionnaire Emesis (PUQE)	[9] Canada, [10] Canada
	McGill nausea questionnaire	[11] Canada
	Rhodes’ scores (RI)	[12] Canada
	Short form – 36 (SF-36)	[13] China
	SF-12, NVP specific QOL	[14] Canada
	NVPQOL, SF-36, SCL90	[15] USA
**Irritable bladder**	Incontinence Impact Questionnaire (IIQ), Urogenital Distress Inventory (UDI)	[6] Netherlands
	Incontinence Questionnaire Short Form (ICIQ-SF)	[16] Turkey
**Back pains**	Nottingham Health Profile (NHP) Disability Rating Index (DRI)	[17] Sweden
	Pregnancy Mobility Index (PMI)	[18] Netherlands
	WHOQOL-BREF	[19] Turkey
**Anxiety and depression**	Finnish modification of SF of the beck depression inventory and anxiety	[20] Finland
	Hospital Anxiety and Depression Scale (HADS)	[21] Sweden
	EPDS, SF-36v2	[22] China
	WHOQOL-BREF, EPDS	[23] Austria

Symon analyzed 32 studies from MEDLINE, CINAHL and BIDS databases that examined the quality of life during pregnancy and after childbirth and concluded that there are not enough specific tools for use in obstetric care. The present specific questionnaires (PUQE, NVP, QOL) are focused more on specific problems in pregnancy (such as nausea and vomiting) rather than on women’s overall well-being and their quality of life [24]. Mogos note that in 57% of the analyzed studies only generic tools are used (most frequently the SF-36 Short-Form Item 36 and SF-12, WHOQoL-BREF *World Health Organization’s Quality of Life Scale - BREF*), while specific tools are used in only 20% (mostly MGI *Mother-Generated Index,* MAPP–QoL *Maternal Perceived Quality of Life*, NVPQoL *Nausea Vomiting Pregnancy Quality of Life*). A combination of generic and specific tools was used in 23% of the examined studies [3].

The aim of our study therefore was to validate the newly designed specific questionnaire and determine if it is sensitive enough to provide an optimal evaluation of the quality of life of women with a normal pregnancy.

We used focus groups to obtain the source materials for the construction of a new specific questionnaire. Results of this pilot study have already been published [7], and therefore we present only briefly the methodology and conclusions. Seven focus groups of pregnant women took place with the main topic being “The effects of pregnancy on the quality of life of a pregnant woman.” Transcripts of focus group recordings were analyzed using the grounded theory of Strauss and Corbin [25]. We described the perception of changes that pregnancy brings, including the impact of these changes on the quality of life, and determined the variables that, from the perspective of pregnant women, significantly affect their quality of life. The main outcome of the pilot was a theoretical model with six variables that affect and describe the quality of life in physiologically pregnant women (see Table 2): preparing for the role of mother, change of values, self-reflection, acceptance of changes, enrichment of life, and sense of responsibility.

**Table 2:** **Structuring the quality of life in pregnancy according to the model of quality of life by **[26] **and by the causal model (taken from: **[7]**)**

	**External quality of life**	**Internal quality of life**
	**(Environment)**	**(Individual)**
**Life chances,**	Family/Home life	Acceptance of changes
**Life opportunities**	Daily life	Change of the value system
	Economic status	
**Result of life**	Enrichment of life	Self-reflection
**Form of life**	Fulfilling the role of mother	Responsibility

## Objective

The aims of the study were threefold: first, to examine the properties of the generic WHOQOL-BREF in a group of pregnant women; second, to examine the properties of the new specific measure, the QOL- GRAV; and finally to compare the results obtained with QOL-GRAV with those obtained with WHOQOL-BREF.

### Sample

The study used a convenience sample of 225 pregnant women in the first trimester of a routine pregnancy. The following criteria was used for selecting participants: women up to the 16^th^ week of a routine pregnancy, when the first trimester data collection took place, willing to take part in the research and to agree with long-term cooperation. The sample of pregnant women was recruited from pregnant women in prenatal care at private gynaecologists in the town Hradec Kralove. Pregnant women presenting for prenatal care were approached and asked to participate in a research study. All participants were volunteers and signed a written informed consent statement prior to taking part in the study.

The mean age of pregnant women at the beginning of the study (in the first trimester) was 29.7 (SD = 4.9). Of these, 190 had planned their pregnancy and 35 had become pregnant unintentionally. Of the total number of women, 96 were pregnant for the first time, 83 for the second time, 29 for the third time and 17 had been pregnant more than three times; 114 women gave birth for the first time, 87 for the second time, 18 for the third time, and 6 for four or more times. Further demographic characteristics are listed in Table 3.

**Table 3:** Sample demographic characteristics

**Education**		**Primary**	**Secondary comprehensive**	**Secondary vocational**	**College**	**Total**
	**n**	12	121	18	72	223
	**%**	*5.4*	*54.3*	*8.1*	*32.3*	*100.0*
**Occupation**		**Employed/self-employed**	**Unemployed**	**Studying**	**Maternity leave**	
	**n**	134	15	7	67	223
	**%**	*60.1*	*6.7*	*3.1*	*30.0*	*100.0*
**Marital status**		**Single**	**Married**	**Divorced**	**Widowed**	
	**n**	80	127	15	1	223
	**%**	*35.9*	*57.0*	*6.7*	*.4*	*100.0*

## Methods

We started with a general questionnaire, the WHOQOL-BREF (its use has been approved by the author of the Czech version [27]). We decided on the World Health Organization Quality of Life generic questionnaire due to our concern with physiological pregnancy, i.e. with the population of healthy women. Moreover, this questionnaire [3] is the most commonly used generic instrument for assessing the quality of life in pregnancy. WHOQOL-BREF contains a total of 26 items grouped into four domains and two separate items evaluating the overall quality of life (Q1) and satisfaction with one’s state of health (Q2). The results are expressed in terms of four domain scores and mean raw scores of two separate items: D1- Physical (7 items), D2 – Psychological (6 items), D3 - Social relations (3 items), D4 - Environment (8 items). The higher the score values, the better the quality of life.

Based on the results of the pilot study we developed a specific questionnaire, the QOL-GRAV. This measure has been designed as a supplement to the WHOQOL-BREF. The questionnaire includes socio-demographic data relating to pregnancy and 12 five-point Lickert items associated with pregnancy that reflect the structure of the four domains of quality of life (physical, psychological, social relations and environment) of the WHOQOL-BREF. The accompanying commentaries, individual items and scaling anchors have the same form as in the WHOQOL-BREF. Some items of the QOL-GRAV (27, 28) are further supplemented by open-ended questions asking for more detailed specifications of changes during pregnancy and coping strategies. Items 27 and 30–32 relate to the first domain of the physical QoL, while the psychological domain is represented by items 28, 29, and 38. The domain of social relations is represented by items 36 and 37, and items 33 and 34 represent the domain of environment (see the list of items in Appendix).

Both questionnaires were filled in by pregnant women during the three trimesters. Data collection lasted from March 2010 to August 2011.

For statistical data analysis, procedures of descriptive and inductive statistics contained in the software package SPSS version 18 were used. For the estimation of reliability we used Cronbach alpha estimates of internal consistency ranging from 0 to 1 with the arbitrary value of .7 used as the minimum satisfactory level of internal consistency for research purposes.

## Results and discussion

### Results

The initial number of 225 pregnant women in the sample was reduced to 219 women in the second trimester due to pregnancy complications and early termination. The sample was further reduced to 204 women in the third trimester.

1. Psychometric characteristics of the Czech version of WHOQOL-BREF in a group of physiologically pregnant women.

We looked at the internal consistency of individual WHOQOL-BREF scales at different trimesters of pregnancy (see Table 4). Table 5 shows the mean scores of domains (D1–D4) and of individual items (Q1, Q2) of the generic questionnaire WHOQOL-BREF.

**Table 4:** Internal consistency of WHOQOL-BREF scales in a group of pregnant women at different times (Cronbach alpha)

**Domains**	***Trimester I***	***Trimester II***	***Trimester III***
**D1 Physical health**	.78	.77	.86
**D2 Psychological**	.73	.74	.75
**D3 Social relations**	.63	.67	.61
**D4 Environment**	.78	.78	.80

**Table 5:** **Descriptive statistics of the domains and individual items of WHOQOL-BREF in trimester** I

**WHOQOL domains**	**N**	**M**	**SD**
**D1 Physical health**	225	3.81	0.56
**D2 Psychological**	225	3.96	0.48
**D3 Social relations**	225	4.17	0.56
**D4 Environment**	225	3.88	0.49
**Q1 How would you rate your quality of life?**	225	4.05	0.63
**Q2 How satisfied are you with your health?**	225	3.97	0.60

**Figure 1:**
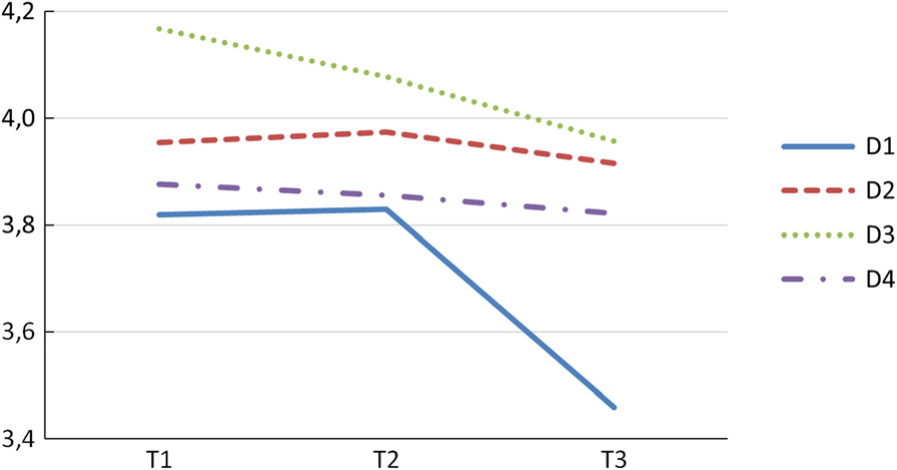
Mean scores for the four WHOQOL-BREF domains over the three trimesters.

The differences between trimesters were tested using repeated-measures ANOVAs. Post-hoc comparisons with Sidak type-I-error correction were used. The physical health (D1) during pregnancy seems to have declined nonlinearly, with the decline appearing to be faster in the second trimester than in the first. In the psychological domain (D2) there is a statistically significant difference between trimesters II and III but the overall trend is fairly flat. In the domain of Social relations (D3) there is a clear downward trend, with the differences being significant between all trimesters. Although the mean scores in the domain of Environment (D4) decrease only slightly, the linear contrast test shows this decrease is also statistically significant (see Figure 1).

In individual items related to overall quality of life (Q1) and satisfaction with health (Q2) no differences between trimesters have been found.

2. Using the Principal Axis Factoring method, analyses of QOL-GRAV items 27 to 38 for each trimester are presented in Tables 6 and 7. According to the screeplots it is possible to distinguish meaningfully three factors in all three waves of data collection (in all three trimesters) with a strong first factor. The first factor is saturated by evaluation items and can be directly related to the quality of life. The second factor expresses how seriously the woman takes her pregnancy. However, it can also be a sign of socially desirable responding (see Table 6). Varimax rotated solutions are more affected by the sample size, with the results being quite different in each trimester (see Table 7).

**Table 6:** QOL-GRAV factor loadings, unrotated solutions

**Item**	**Trimester I**			**Trimester II**			**Trimester III**		
	**1**	**2**	**3**	**1**	**2**	**3**	**1**	**2**	**3**
**A27**	**.53**	-.26	**.43**	**.56**	.10	.16	**.64**	.11	-.04
**A28**	**.52**	.14	.06	**.55**	.09	-.01	**.63**	-.02	-.02
**A29**	.08	**.62**	-.07	.22	**.50**	.01	**.31**	**.44**	-.12
**A30**	**.50**	.09	-.04	**.51**	.16	-.11	**.57**	.10	.12
**A31**	.26	**.32**	-.11	**.42**	**.36**	-.27	**.38**	.05	.27
**A32**	**.48**	**.37**	**-.34**	**.51**	**.31**	-.29	**.56**	.20	**.43**
**A33**	-.26	.09	.28	-.20	.28	.25	-.27	.25	-.11
**A34**	-.02	**.61**	.15	-.02	**.56**	**.34**	.20	**.52**	-.22
**A35**	**.43**	.15	**.38**	**.36**	.08	**.35**	**.44**	.09	**-.39**
**A36**	**-.42**	.17	**.37**	**-.47**	**.36**	.07	**-.39**	**.38**	.10
**A37**	**-.60**	.23	.03	**-.64**	.26	-.06	-.25	.24	-.03
**A38**	**-.71**	**.31**	-.06	**-.59**	**.39**	-.29	**-.57**	**.45**	.20

Note. All loadings above .3 in bold type.

**Table 7:** QOL-GRAV factor loadings, Varimax rotated solutions

**Item**	**Trimester I**			**Trimester II**			**Trimester III**		
	**1**	**2**	**3**	**1**	**2**	**3**	**1**	**2**	**3**
**A27**	**.69**	-.02	-.01	**.48**	**.33**	.05	**-.33**	**.39**	**.41**
**A28**	**.43**	-.24	.23	**.30**	**.40**	-.05	**-.41**	**.37**	**.30**
**A29**	-.02	.06	**.63**	.04	**.43**	**.33**	.10	.21	**.50**
**A30**	**.35**	**-.32**	**.19**	**.27**	**.47**	**-.06**	**-.24**	**.47**	**.27**
**A31**	.11	-.16	**.39**	.05	**.62**	.01	-.13	**.45**	.05
**A32**	.13	**-.46**	**.51**	.12	**.65**	-.06	-.08	**.72**	.13
**A33**	-.01	**.39**	-.01	-.08	-.09	**.41**	**.32**	-.18	.09
**A34**	.05	.28	**.56**	.03	.15	**.64**	.20	.09	**.56**
**A35**	**.57**	.05	.17	**.45**	.09	.20	**-.31**	.00	**.50**
**A36**	-.09	**.58**	.02	**-.42**	-.12	**.41**	**.55**	-.07	-.01
**A37**	**-.45**	**.46**	.09	**-.59**	-.21	.29	**.32**	-.12	.05
**A38**	-.59	**.47**	.17	**-.72**	-.01	.24	**.74**	-.09	-.12

Note. All loadings above .3 in bold type.

3. Properties of the pregnancy QoL scale.

Our aim was to construct one or more unidimensional scales from the available Pregnancy-QoL items, the validity of which could be explored in further research. Based on exploratory factor analyses, we decided conservatively to construct only one evaluation scale consisting of 9 items with stable high loadings on the first factor (27, 28, 30, 31, 32, 35, 36r, 37r and 38r). The scale value is computed as the mean of responses to all included items. Table 8 presents descriptive statistics for this scale. Low scale values mean a high quality of life and an absence of problems. Scales suggested by the second and third factors are less theoretically supported and the number of items was not high enough to construct an internally consistent scale when considering the level of inter-item correlations.

**Table 8:** Descriptive statistics of pregnancy QoL scale in each trimester

**Pregnancy QoL**	**N**	**Min**	**Max**	**M**	**SD**	**Cronbach alpha**
**Trimester I**	225	1.00	3.67	2.19	.51	.72
**Trimester II**	219	1.00	3.78	2.16	.48	.74
**Trimester IIII**	204	1.00	3.89	2.29	.50	.75

Whereas in the first two trimesters the mean value of Pregnancy QoL remains the same, in the third semester the mean value slightly increases (Cohen *d* = .2; *p* < .01). The correlations between trimesters remain quite high (see Table 9) and suggest long-term QoL stability.

**Table 9:** Correlations of pregnancy QoL scale between trimesters

**Pregnancy QoL**	**Trimester II**	**Trimester III**
**Trimester I**	.76	.62
**Trimester II**		.71

Note. All correlations p < .01.

When we look at the intercorrelations between our new Pregnancy QoL scale and the WHOQOL-BREF domain scales (see Table 10) their range in concurrent measurements runs from .44 to .69.

**Table 10:** Correlation of the domains of the WHOQOL questionnaire and the pregnancy scale in individual trimesters

	**Pregnancy QoL scale**		
**WHOQOL-BREF**	**Trim. I**	**Trim. II**	**Trim. III**
**Trimester I**			
**Physical health**	.64	.49	.41
**Psychological**	.54	.52	.49
**Social relations**	.46	.37	.29
**Environment**	.44	.47	.34
**Trimester II**			
**Physical health**	.51	.59	.46
**Psychological**	.48	.57	.51
**Social relations**	.36	.44	.45
**Environment**	.37	.50	.40
**Trimester III**			
**Physical health**	.53	.55	.69
**Psychological**	.51	.60	.62
**Social relations**	.38	.45	.53
**Environment**	.37	.45	.46

Note. All correlations p < .01. Due to the different orientations of Pregnancy QoL scale and WHOQOL scales all correlations are negative; the negative sign has been removed for clarity.

## Discussion

The internal consistencies of WHOQOL-BREF domain scales in this study are similar to those found in a sample of healthy individuals of a selected Prague population sample in the age group (18–59) studied when validating the Czech version of the WHOQOL-BREF [27]. The only item showing a slight difference was item 4 (q4), which asks about the need for medical care. We think it is because most pregnant women see pregnancy as normal condition for which they do not think any special medical care is needed to help them with daily, routine living. During pregnancy, the assessment of quality of life (Q1) and satisfaction with one’s state of health (Q2) does not change, but the assessment of individual domains varies, especially social relationships (D3) and the environment (D4), where clearly declining trend can be noticed. The decline may be related to the increasing isolation of pregnant women in proportion to gestational age. Trimester III has the lowest mean, the highest mean is in trimester II.

A research team led by Haas examined changes in the health status of women both during pregnancy and after childbirth [28]. Using selected items from the MOS, SF-36 and CES-D they found that substantial changes (affecting the quality of life) occurred in trimester III, when the bodily functions suffered and their improvement occurred a lengthy time after birth (in 8 to 12 weeks). The prevalence of psychological problems increased in the third trimester as well. We also found that the lowest quality of life in trimester III was connected with the lowest mean score in the domainof Physical health (physical functions) and Psychological (mood changes). Fernandes and Vido identified the quality of life of pregnancy during the three trimesters of pregnancy using the QLI *Quality of Life Index *[29]. They found a statistically significant difference among pregnant women in the first and second trimester. Pregnant women reported higher quality of life in the first trimester than in the second one. A significant difference between trimester II and III and between trimester I and III has not been found. In our study, a statistically significant difference between all the three trimesters in the domains D1-D4 was found, but there was no difference between trimesters in separate items of quality of life (Q1) and satisfaction with health condition (Q2).

An interesting comparison is presented by the Brazilian scientific team led by Vallim, who studied the effects of water exercises on quality of life of pregnant women during routine pregnancy [30]. Vallim used the WHOQOL-BREF questionnaire and, as in our study, respondents were asked to fill it in at three different times (at the beginning of the pregnancy, in the 28th week, and in the 36th week). Although their findings show that water exercises do not affect the quality of life of pregnant women, it is possible to compare the mean scores of the overall satisfaction with health (Q2) and the quality of life (Q1), which (as in our study) do not change during the trimesters. Similar results are found even in the domain of Physical health (D1) and show a slightly decreasing trend as well. Unlike in our study, the domain of Environment (D4) increases and the highest mean score was achieved in the 36th week of pregnancy. Pregnant women in Brazil do not seem to suffer from isolation at the end of pregnancy in the same way as Czech women. It probably results from both the specific set of pregnant women who underwent special water exercises as a part of prenatal care and antenatal preparation, and from the culturally different environment.

The new QOL-GRAV pregnancy scale consists of 9 items. It has satisfactory internal consistency and variability and expresses the degree of specific experiences during pregnancy. The interpretation of values of the quality of life according to this QOL-GRAV scale means that the lower the score, the higher the quality of life. The mean scores of the QOL-GRAV scale are generally lower than in the generic WHOQOL-BREF questionnaire. Considering the internal consistencies of QOL-GRAV and WHOQOL-BREF, the pattern of correlations between the QOL-GRAV and WHOQOL-BREF scales suggest that we could argue for the concurrent validity of QOL-GRAV. It appears to correlate with the WHOQOL-BREF score and corresponds to the individual scales/domains of WHOQOL-BREF. We could thus think of QOL-GRAV as an optional domain of WHOQOL-BREF.

## Conclusions

We developed a new specific QOL-GRAV questionnaire to evaluate the quality of life of women with a normal pregnancy. It has 9 items and its psychometric characteristics are satisfactory. Our results suggest that both the general quality of life of pregnant women and the specific quality of life of pregnant women are related but different. In both questionnaires, the highest quality of life was in the second trimester and the lowest in the third trimester. Similar results were also reached in the above mentioned studies in which generic questionnaires were used.

Although the generic WHOQOL-BREF questionnaire can also be used for evaluating the quality of life of healthy pregnant women, the specific questionnaire provides an opportunity to capture more sensitively and accurately the degree of specific experiences during physiological pregnancy substantially affecting the quality of life of pregnant women.

The expected contribution of this simple questionnaire may be seen in its use in prenatal care. Knowledge of how a particular woman evaluates the quality of her life during a routine pregnancy could lead to increased efficiency of care for pregnant women and to an increase in their well-being.

We studied pregnant women aged 19 to 42. These women mostly planned their pregnancy, were employed, had secondary or university education, were primiparas, were willing to cooperate and were in optimal relationships. All pregnant women came from majority Czech population. The results of the study were determined by the Czech health care system, e.g. compulsory health insurance and a positive attitude to prenatal care and participation in prenatal screening.

In the following study it would be helpful to see how the specific questionnaire works with pregnant women of higher and lower age, of socioeconomic, cultural and ethical influences (minority groups, the socially disadvantaged and women who do not attend prenatal care). In addition, it would be beneficial to see if the specific QOL-GRAV scale works in other countries.

## Endnotes

Items marked r 36–38 are reverse coded.

## Appendix

### Items of the QOL-GRAV questionnaire

A27 To what extent do you feel that your physical changes associated with this pregnancy do not allow you to do what you need?

A28 To what extent do you feel that your psychological changes associated with this pregnancy do not allow you to do what you need?

A30 How worried are you about not being able to handle household chores?

A31 How worried are you about carrying out the pregnancy successfully?

A32 How worried are you about not being able to handle labor and delivery?

A35 Have you been forced to cut down on your physical activity during this pregnancy?

A36r How satisfied are you with your partner now?

A37r How satisfied are you with your social life now?

A38r How satisfied are you with how you manage to adapt to this pregnancy?

## Competing interests

The authors declare no conflicts of interests.

## Authors’ contributions

EV participated in the desing of the study, carried out the questionnaire inquiries and drafted the manuscript. SJ performed the statistical analysis and edited the final manuscript. JM participated in the desing of the study and helped to draft the manuscript. MM participated in the coordination of the study. All authors read and approved the final manuscript.

## Authors’ information

Eva Vachkova, MS, RM - head of Division of Nursing

Stanislav Jezek, MS, PhD - researcher at Institute for Research of Children, Youth and Family

Jiri Mares, Prof., Dr., CSc. - deputy head of Department of Social Medicine

Marketa Moravcova, MS, RM - head of Department of Midwifery and Health and Social Work.
